# Supplementary material for: In Silico Characterization and Analysis of Clinically Significant Variants of Lipase-H (LIPH Gene) Protein Associated with Hypotrichosis
Source: Pharmaceuticals (Basel). 2023 May 29;16(6):803. doi: 10.3390/ph16060803 (PMC10302509; doi:10.3390/ph16060803)
Supplement: Supplementary file 1 [file pharmaceuticals-16-00803-s001.zip › pharmaceuticals-2364388-supplementary.pdf]

Supplementary Files

Supplementary Table S1. Details of the 126 missense mutations, that are identified as deleterious by all the five tools.

Variant ID	vf_allele	Alleles	AA	AA coord	SIFT	PolyPhen	REVEL	MetaLR	Mutation Assessor
rs1001254325	C	T/C	T/A	26	0	0.967	0.53	0.884	0.905
rs538672593	G	A/G	F/S	30	0.01	0.781	0.5	0.839	0.879
rs777707092	T	C/T	A/T	33	0.11	0.964	0.502	0.833	0.755
rs906946330	A	C/A	G/C	36	0	0.696	0.797	0.846	0.931
rs371942576	A	G/A	T/M	37	0	0.998	0.686	0.861	0.828
rs1560167771	C	T/C	T/A	37	0.03	0.983	0.683	0.805	0.602
rs61749467	T	C/T	V/M	41	0.06	0.995	0.581	0.76	0.851
rs1349689376	G	A/G	V/A	41	0	0.977	0.812	0.885	0.818
rs754715516	C	A/C/G	L/R	45	0	0.999	0.951	0.926	0.991
rs754715516	G	A/C/G	L/P	45	0	0.999	0.949	0.932	0.968
rs751010107	A	G/A	T/I	47	0	0.999	0.772	0.914	0.977
rs1560167737	G	T/G	T/P	47	0.01	0.999	0.853	0.9	0.966
rs567746197	T	C/T	C/Y	53	0	1	0.716	0.851	0.805
rs777122848	T	C/T	A/T	54	0	0.983	0.562	0.822	0.676
rs1158464387	A	G/A	H/Y	76	0	1	0.96	0.962	0.986
rs1560167651	A	C/A	G/V	77	0	1	0.969	0.988	0.996
rs747880338	C	G/A/C	F/L	78	0.01	0.506	0.746	0.848	0.913
rs150941910	T	C/T	R/K	79	0	0.98	0.673	0.813	0.585
rs1176079211	C	T/C	T/A	81	0.01	0.755	0.563	0.816	0.794
rs765837711	G	A/G	L/S	91	0	0.959	0.775	0.878	0.886
rs1577682011	T	A/T	V/E	92	0.01	0.898	0.711	0.838	0.831
rs757899554	G	A/G	L/P	96	0	0.995	0.789	0.907	0.989
rs764563463	A	T/A	D/V	100	0	0.989	0.835	0.922	0.988
rs267607219	G	A/G	W/R	108	0	1	0.952	0.932	0.995
rs1449651719	G	T/G	N/H	109	0	0.997	0.823	0.829	0.735
rs370653261	A	C/A/T	R/L	110	0	0.943	0.587	0.832	0.822
rs1173584831	T	C/T	A/T	112	0	0.999	0.882	0.908	0.919
rs1256399071	A	G/A	T/I	113	0	0.952	0.683	0.82	0.631
rs199508534	A	G/A	T/I	114	0	0.984	0.529	0.84	0.773
rs1269981216	T	C/T	A/T	120	0	0.982	0.716	0.922	0.92
rs201061198	G	A/G	S/P	121	0.02	0.555	0.545	0.83	0.914
rs762974986	T	C/T	A/T	128	0	0.994	0.786	0.887	0.902
rs1577681837	A	C/A	L/F	131	0	0.998	0.833	0.867	0.896
rs768489997	G	A/G	I/T	135	0	0.987	0.947	0.878	0.856
rs1369025550	G	C/G	D/H	136	0.06	0.928	0.62	0.867	0.763

rs1553824000	T	C/T	G/E	142	0	0.999	0.729	0.901	0.921
rs770550405	C	T/C	Y/C	149	0	0.986	0.846	0.872	0.936
rs770313843	T	C/T	G/R	152	0	1	0.984	0.995	0.996
rs748606060	C	G/C	L/V	155	0	1	0.918	0.907	0.916
rs1368717012	G	C/G	G/A	156	0.02	0.996	0.962	0.997	0.949
rs1217158658	T	C/T	G/R	164	0	1	0.973	0.978	0.994
rs765279202	T	G/T	L/M	171	0	0.887	0.588	0.896	0.892
rs762002438	A	C/A	R/I	173	0	0.998	0.952	0.972	0.993
rs1320917299	T	C/T	G/S	176	0.02	0.998	0.887	0.93	0.942
rs1484869809	G	C/G	D/H	178	0	1	0.968	0.974	0.996
rs766041291	A	G/A/C	P/S	179	0	1	0.973	0.956	0.987
rs766041291	C	G/A/C	P/A	179	0.02	1	0.926	0.909	0.861
rs138900382	C	G/C	A/G	180	0	1	0.938	0.941	0.979
rs536408544	T	C/T	A/T	180	0	1	0.961	0.918	0.935
rs1185160123	T	C/T	G/D	181	0	1	0.905	0.875	0.908
rs1008933418	A	G/A	P/S	182	0	1	0.915	0.914	0.97
rs1251506312	A	G/A	P/L	182	0	1	0.891	0.892	0.898
rs769118659	T	C/T	R/K	192	0.01	0.98	0.901	0.876	0.8
rs747561558	C	T/C	D/G	194	0.01	0.996	0.897	0.891	0.919
rs746244349	G	T/G	Q/P	199	0	0.956	0.834	0.833	0.879
rs1433834243	C	A/C	F/L	200	0.01	0.914	0.934	0.929	0.824
rs778994315	C	T/C	D/G	202	0	0.999	0.965	0.948	0.992
rs1355676735	T	C/T	D/N	202	0	0.999	0.902	0.952	0.98
rs372971860	T	C/T	V/I	203	0.04	0.951	0.617	0.832	0.662
rs778051082	C	T/C	H/R	205	0	1	0.977	0.972	0.995
rs565980022	A	G/A	H/Y	205	0	1	0.974	0.961	0.932
rs1560163575	G	A/G	S/P	206	0	0.985	0.875	0.878	0.949
rs756287038	G	C/A/G	D/H	207	0	0.999	0.944	0.914	0.935
rs756287038	A	C/A/G	D/Y	207	0	0.999	0.911	0.892	0.808
rs1315409277	G	A/G	L/P	211	0	0.994	0.945	0.918	0.97
rs369104457	G	A/G	L/S	217	0	0.893	0.715	0.818	0.682
rs1287841272	T	C/T	G/R	218	0	1	0.966	0.955	0.995
rs866749761	T	C/G/T	G/E	218	0	1	0.956	0.955	0.995
rs866749761	G	C/G/T	G/A	218	0	1	0.893	0.903	0.798
rs760042144	G	A/G	I/T	220	0	0.994	0.869	0.825	0.758
rs371437625	G	A/G	Y/H	223	0	0.999	0.946	0.932	0.983
rs1453368208	A	G/A	P/L	224	0	0.997	0.839	0.898	0.904
rs760141096	A	T/A	N/I	225	0	1	0.901	0.951	0.996
rs771383305	T	C/T	G/E	226	0	0.999	0.966	0.977	0.977
rs1405858753	C	T/C	D/G	229	0.01	0.793	0.887	0.825	0.683
rs1177415706	C	G/C	P/R	231	0	1	0.95	0.973	0.936

rs1560161495	T	C/T	G/R	240	0.01	0.997	0.892	0.921	0.905
rs763397105	C	T/C	Y/C	243	0	0.997	0.86	0.872	0.904
rs201249971	T	A/T	C/S	246	0	1	0.956	0.617	0.986
rs201868115	T	G/T	H/N	248	0	0.994	0.924	0.942	0.995
rs762143873	A	C/A/T	R/M	250	0.04	0.633	0.691	0.901	0.798
rs141948236	A	G/A	S/F	251	0	0.992	0.95	0.934	0.982
rs960010245	G	C/G/T	V/L	252	0	0.588	0.509	0.762	0.685
rs1284573868	T	A/T	Y/N	255	0	0.981	0.933	0.925	0.976
rs768740653	G	T/G	Y/S	255	0	0.962	0.905	0.913	0.976
rs1445790092	G	A/G	S/P	258	0	0.994	0.913	0.945	0.981
rs770909511	G	A/G	L/P	259	0	0.995	0.94	0.593	0.951
rs755886556	T	G/A/T	A/E	267	0	0.918	0.723	0.635	0.961
rs780714833	C	A/C	Y/D	268	0	0.985	0.694	0.886	0.927
rs1031272901	A	G/A	P/S	269	0.02	0.946	0.789	0.859	0.712
rs1208678501	T	C/T	C/Y	270	0	0.99	0.898	0.713	0.994
rs1274322632	A	G/A	S/F	272	0	0.997	0.911	0.912	0.955
rs754454368	C	T/C	Y/C	276	0	0.992	0.827	0.91	0.965
rs1235968992	T	C/T	G/D	279	0	0.994	0.872	0.87	0.832
rs1410541813	G	C/G	C/S	281	0	0.999	0.969	0.596	0.952
rs770837697	A	G/A	P/S	293	0	1	0.82	0.834	0.662
rs141115870	G	C/G	G/R	296	0	0.997	0.97	0.979	0.977
rs769679909	C	T/C	Y/C	297	0	0.992	0.688	0.898	0.95
rs776331827	G	T/C/G	D/A	300	0.09	0.542	0.635	0.875	0.86
rs776331827	C	T/C/G	D/G	300	0.06	0.719	0.643	0.857	0.848
rs879101399	A	C/A	W/L	302	0	0.964	0.741	0.788	0.738
rs950306164	G	A/G	L/P	306	0	0.995	0.609	0.803	0.753
rs1273377485	G	C/G	A/P	321	0.08	0.936	0.515	0.816	0.755
rs757769060	T	G/T	P/Q	325	0	0.998	0.9	0.971	0.988
rs1433870719	G	A/G	I/T	335	0.02	0.641	0.62	0.761	0.808
rs1265005542	T	C/T	G/R	345	0	0.997	0.798	0.842	0.856
rs1157940221	C	G/C	S/C	361	0.04	0.928	0.67	0.787	0.792
rs768835064	C	T/C	Y/C	373	0	0.96	0.721	0.795	0.832
rs533334084	A	G/A	L/F	379	0.06	0.908	0.633	0.805	0.862
rs878908043	A	C/A	L/F	394	0.02	0.951	0.52	0.743	0.832
rs781204756	T	A/T	F/I	396	0.12	0.566	0.606	0.75	0.528
rs145905764	A	G/A	T/I	398	0	0.786	0.619	0.758	0.828
rs1391731106	A	T/A	R/W	416	0.02	0.924	0.633	0.75	0.758
rs373209531	A	G/A	P/L	421	0.01	0.815	0.556	0.755	0.828
rs146753303	A	G/A	R/W	428	0.02	0.998	0.694	0.818	0.853
rs764473746	G	C/G/T	R/P	428	0	0.995	0.848	0.838	0.853
rs764473746	T	C/G/T	R/Q	428	0.01	0.975	0.552	0.81	0.853

rs775881352	T	A/G/T	D/E	430	0	0.983	0.573	0.743	0.847
rs1219851021	G	A/G	V/A	432	0	0.692	0.611	0.732	0.808
rs759994648	A	C/A/G/T	V/F	432	0	0.938	0.672	0.806	0.808
rs376062316	T	C/G/T	E/K	435	0.05	0.967	0.803	0.818	0.856
rs376062316	G	C/G/T	E/Q	435	0.02	0.882	0.658	0.845	0.772
rs1040056355	A	T/A	N/I	436	0	0.923	0.59	0.769	0.832
rs1427644457	A	T/A/C	I/F	444	0.02	0.851	0.563	0.792	0.812
rs779862632	G	C/G	C/S	446	0	1	0.819	0.834	0.862
